# Collagen-Composite Scaffolds for Alveolar Bone and Dental Tissue Regeneration: Advances in Material Development and Clinical Applications—A Narrative Review

**DOI:** 10.3390/dj13090396

**Published:** 2025-08-29

**Authors:** Natesan Thirumalaivasan

**Affiliations:** Department of Periodontics, Saveetha Dental College and Hospitals, Saveetha Institute of Medical and Technical Sciences (SIMATS), Saveetha University, Chennai 600077, Tamil Nadu, India; natesant.sdc@saveetha.com or thirumalaivasan.nk@gmail.com

**Keywords:** collagen-based biomaterials, guided tissue regeneration (GTR), dental tissue engineering, collagen scaffolds, biocompatibility and bioactivity, 3D bioprinting, hydroxyapatite (HA) and β-TCP composites, collagen crosslinking, stem cell therapy in dentistry, periodontal tissue engineering

## Abstract

**Background/Objectives:** The use of collagen-based scaffolds in dentition tissue engineering has gained significance and importance in the field as they are structurally equivalent and biologically compatible with the native extracellular matrix (ECM). In this review, collagen-composite scaffolds for pulp, alveolar bone, and periodontal regeneration are analyzed in terms of materials, fabrication techniques, and clinical outcomes. **Methods:** Recent developments in collagen scaffolds are highlighted in this review, with a focus on type I collagen due to its structural strength and arginine–glycine–aspartic acid (RGD) motifs, which promote cell adhesion and differentiation. Composite materials, freeze-drying, electrospinning, and 3D bioprinting, which are used to improve the functionality of the scaffold, are key developments. **Results:** This review shows progress in collagen-based scaffolds for restoring dental tissues, such as dentin, gingival tissue, or bone, in humans. Electrospinning and 3D bioprinting are new manufacturing techniques that enhance the functionality of scaffold devices, and incorporating bioactive molecules increases the regenerative capacity; however, stability and long-term efficacy are still problems. **Conclusions:** Although they have a lot of potential, collagen-composite scaffolds face challenges like rapid degradation and limited mechanical strength. To make long-lasting, tailored dental regeneration therapies feasible, future research needs to improve smart biomaterials, gene delivery, and personalized designs for dental regenerative therapy.

## 1. Introduction

Dentin, pulp, periodontal ligament (PDL), cementum, and alveolar bone are dental tissues needed for proper dental function and health. However, these tissues can easily be affected by several problems, such as dental decay, physical causes of damage, periodontal diseases, and surgical treatments. Using traditional restorations repairs damaged teeth, but they do not always effectively replicate the complex nature and functional unity of actual tooth tissues [1]. Over time, some old amalgam and ceramic materials can erode, become illegible, fail to integrate properly, and sometimes expose patients to problems like increased caries and inflammation. It is very clear that developing new strategies is necessary to improve reparative dentistry and allow the body to help itself heal.

Due to its use of biomaterials, cells, and molecules, tissue engineering now plays a key role in regenerative dentistry by imitating how the body helps tissues recover. To support this method, researchers create scaffolds that temporarily function as the ECM to help cells attach, divide, and change shape for proper tissue development and the restoration of their functions [2]. A perfect scaffold for dental tissue engineering must be biocompatible, biodegradable, as strong as the ECM, and organized in the same way as natural oral tissue. Furthermore, it should encourage blood vessel growth and attachment to neighboring tissues to ensure durability. Collagen matrices have become popular as they share similar biochemical, structural, and functional aspects with dental ECM components [3].

The majority of the organic matter found in dentin and the periodontal ligament is collagen type I, as it makes up around 90% of their material. Collagen gains strength from its triple-helix structure, and at several levels of organization, it remains strong and flexible. Besides supporting structures, collagen contains parts that attract integrins, helping cells adhere to the matrix, and triggering important functions within the cell for tissue formation and repair. Additionally, collagen’s water-attracting properties enable nutrients to travel and be exchanged more easily within the scaffold environment, ensuring that cell life and function are properly maintained during the regeneration process [4,5]. These qualities make collagen an ideal biomaterial scaffold for use in dental GTR.

Still, the limitations of native collagen scaffolds include low resistance to force and a swift reactivity to enzymes compared to dental mineralized tissues. As a result, they are limited in use, for example, during the regeneration of alveolar bone or supporting the periodontal tissues of teeth. For this reason, it has become common to add hydroxyapatite (HA), β-tricalcium phosphate (β-TCP), PLGA, and functional molecules to collagen-based materials to make them stronger and determine how fast they decay. Also, researchers have used chemical and physical methods to repair collagen fibrils, increase the lifespan of scaffolds, and adjust their microstructure to guide cells properly [6,7,8]. These types of modifications have increased the usefulness of collagen biomaterials in various dental tissue engineering applications.

Collagen scaffolds have been improved by new fabrication technologies. Freeze-drying produces collagen sponges with many pores and connections that are easy for cells and blood vessels to enter, while electrospinning is used to make nanofibres that copy the ECM’s natural fibrils. The use of three-dimensional (3D) bioprinting enables researchers to precisely shape the scaffolds, arrange cells on them, and incorporate bioactive factors, which makes it possible to create constructs that match a patient’s anatomy. When collagen self-assembles and gels to form hydrogels, these can be administered in tiny injections to increase the capacity of stem cells and growth factors to repair pulp and periodontal regions [9]. All of these approaches help the scaffold replicate the active and multidimensional microenvironment needed for successful dental tissue regeneration.

Collagen-made biomaterials show great promise for use in clinical settings. When placing collagen scaffolds containing dental pulp stem cells and certain growth factors, tissue similar to dental pulp has formed, accompanied by odontoblast cell growth, which is necessary for reparative dentin formation. Regarding periodontal tissue engineering, membranes filled with collagen, typically called GTR barriers, are often chosen to promote periodontal ligament cell growth and prevent epithelial cell growth down into the tissues. These special scaffolds support growth of the PDL, cementum, and alveolar bone to restore the attachment of soft and hard tissues. Also, as enamel itself does not regenerate, biomimetic innovations guide the dentin and enamel to grow again by using collagen as a pattern [10]. Thus, collagen is important for helping with regeneration in many fields of dentistry.

**Figure 1:**
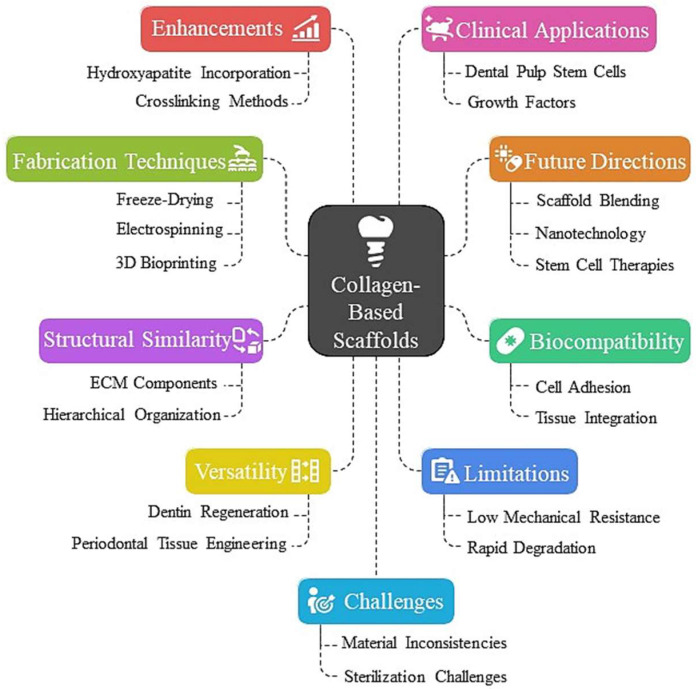
Collagen-based scaffolds for dental tissue engineering-innovations: structural analogies and biocompatibility.

Even so, a few issues stand in the way of using collagen-based biomaterials in medicine. Differences in natural collagen can result in inconsistency in how collagen behaves and acts. Even though immunogenic reactions hardly ever happen, they may occur depending on the source and purification of the collagen. Collagen and bioactivity are usually affected by common sterilization techniques, so it is necessary to find safer sterilization approaches [11]. Besides being broken down too quickly by the body, such scaffolds cannot withstand the force of chewing. It is important to keep researching hybrid scaffolds and new crosslinking methods and to include smart biomaterials in such systems that can react to changes in the body to keep the scaffold effective throughout healing (Figure 1).

For future progress, collagen-based dental tissue engineering will mostly focus on blending scaffolds, nanotechnology, growth factor delivery, and stem cell treatments. Bioactive glass and silver nanoparticles provide antimicrobial action and improve bone growth, and the slow and constant release from encapsulated biomolecules makes sure these effects are sustained. With methods such as 3D bioprinting and electrospinning, it is possible to create scaffolds with unique features meant for specific defect repair. Including gene therapy with collagen scaffolds may boost the recovery process inside the body. Furthermore, using collagen materials that react to external changes could make them fit each patient’s requirements better. With these advances, regenerative dentistry now relies on collagen biomaterials as its main innovations.

## 2. Collagen as a Biomaterial

### 2.1. Composition and Structure

The structural protein known as collagen is present in the human body at a level of about 30% of total protein, indicating how important it is in many biological activities. Of all the types of collagen, Type I is the most common in dental tissues, making up almost all of the organic part of both dentin and the periodontal ligament (PDL). Collagen is clearly key in preserving the strength and proper functioning of the teeth. Molecularly, collagen is twisted into a special formation where three left-handed polyproline II helices are wound to form a right-handed superhelix [12]. This kind of assembly contains multiple repeats of Gly-X-Y amino acid segments, where glycine’s side chain bends easily inside the helix and proline and hydroxyproline play a supportive role through their bonds inside the structure.

Collagen fibers are organized from tiny triple twists to larger fibrillar groups and end up in a well-structured extracellular matrix (ECM). The fact that the vasculature comes in multiple sizes means it is stronger and more flexible and it also forms a microenvironment that is similar to the structure of real tissue. The network structure of fibers is made more stable by crosslinks made naturally by lysyl oxidase enzymes which increase the strength of the collagen matrix [13]. Apart from giving support, collagen has internal parts, especially RGD and other integrin-binding motifs, which assist cells in staying attached to the matrix. They control cell adhesion, movement, reproduction, and changes by connecting cell receptors, mainly integrins, which activates important signaling pathways for the growth and upkeep of tissues [14]. Because of this, collagen biologically influences cells and their activities instead of only supporting their structure.

Collagen’s affinity for water helps it function as a biomaterial by allowing the easy transport of vital substances, gases, and wastes in the scaffold which are all necessary for cell survival during tissue regeneration. It is important for the environment inside the cell and for cell functions like enzymes to be well hydrated. Yet, aside from its many benefits, native collagen also shows a few disadvantages as a material for scaffolding. It lacks the needed toughness to stand alone due to its low stiffness and quick removal when exposed to MMPs, which is why it is not good for hard, heavily stressed areas in the mouth like the alveolar bone or affected gingival tissue [15]. As a result, it is necessary to create collagen composites through engineering and stabilize them using chemical or physical methods, adding in agents such as hydroxyapatite or synthetic polymers to achieve better strength and manage the rate of breaking down of the material. To conclude, its formation, shape, structure, and bioactivity confirm collagen as a remarkable choice for applications in dental tissue engineering. Collagen’s ability to resemble the natural extracellular matrix and its helpful biochemical and biomechanical properties are what makes collagen-based scaffolds excellent for dental tissue repair and restoration.

### 2.2. Biological Properties

As collagen shows great biocompatibility, it is frequently used in dental tissue engineering. Being part of the natural body matrix, type I collagen is quickly recognized by cells, which helps to prevent local immune reactions and decreases inflammation. The lack of an immune response due to this protein is to the way the molecule is made in nature and to how it is separated from other components. Because biocompatibility is provided, cells can stick to the matrix, migrate, grow more, and change into different types which are crucial for proper tissue repair [2,16]. Especially, collagen enables dental pulp stem cells, periodontal ligament fibroblasts, and osteoblast cells to properly adhere and function in dental tissues, which helps the scaffold play an active role in cell signaling and tissue development (Table 1).

#### 2.2.1. Biodegradability

Collagen scaffolds are special because they can be enzymatically broken down in a controlled manner. Proteolytic enzymes produced by cells in the area promote the breakdown of collagen, coming from collagenases, MMPs, and other substances. Due to biodegradation, the scaffold disappears gradually, helping new native extracellular matrix appear in the area. For the wounds to recover, degradation needs to occur at the same rate as tissue formation to help keep the structure from breaking and still allow new tissue growth [17,18]. Even though unmodified collagen scaffolds are useful in tissue regeneration, their quick degradation can make them unsuitable for delivering load-bearing support in dentistry. Because of this, techniques like adding crosslinks and mixing materials are used to slow down biodegradation, which allows the scaffolds to be present during vital parts of tissue repair.

#### 2.2.2. Bioactivity

This activity of tissue stimulation provided by collagen means that it is the most suitable form of biomaterial scaffold to use in dental tissue engineering. It is inherently possessing integrin-binding sites, primarily the RGD peptide fragments binding with integrin cell surface receptors. They facilitate cell attachment, and process signaling amidst cells and cell activation inside the cell, culminating in processes influencing proliferation, cell differentiation, and production of extracellular matrix. Due to these bioactive motifs, collagen is used to help sculpture cells into dentin, pulp, periodontal ligament, and alveolar bone formers. In fact, it is interesting to note that to replicate the cell interactive features of collagen, synthetic scaffolds are often designed to resemble the RGD sequences, thus highlighting their importance in the designing of biomaterial. In addition, spatial arrangement and density of RGD peptides could be regulated to a certain degree of perfection to facilitate the production variable cellular reactions to improve the outcome of tissue regeneration [19]. To further improve its role, collagen can be modified with either growth factors (i.e., BMPs and VEGF) or antimicrobial agents, resulting in customized scaffolds for regenerative medicine.

#### 2.2.3. Hydrophilicity

Collagen performs well as a scaffold in the body because it has inherent attraction to water. As it is hydrophilic, collagen enables the body to absorb and hold aqueous fluids, making the area around the cells suitable for smooth nutrient movement, breathing, and waste disposal. As a result, cells remain active and the scaffold performs its functions during tissue regeneration. In addition, remaining hydrated keeps enzymes active while supporting the ability of cells to shape and reshape themselves. Using freeze-drying and crosslinking in the manufacturing process can change the porosity and water content of collagen matrices, which helps vascular infiltration and attachment to nearby tissues [20]. The use of collagen that dissolves in water and sends bioactive signals makes collagen scaffolds like the natural dental ECM helpful in complex regenerative processes.

**Table 1:** Information on the essential substances and processes used to create collagen scaffolds for dental tissue regeneration is given below. It helps explain, in a step-by-step way, what is essential for making and using scaffolds.

Material	Source	Physical Properties	Chemical Properties	Applications	Ref.
Collagen Type I	Collagen (animal/human), HA (bone-derived) and PLGA, PCL (lab-made for consistency).	Porosity, elasticity, tunable degradation rate.	Dictates bioactivity, immune response, and degradation.	Periodontal tissue and bone regeneration. Guided tissue regeneration scaffolds (GTR). Regeneration of dental pulp.	[21]
Hydroxyapatite (HA)	Natural bone, synthetically produced (Sol–gel method).	Hard, crystalline, bioactive ceramic, highly porous.	Calcium phosphate (Ca_10_(PO_4_)_6_(OH)_2_).	Improves bone regeneration, enhancing collagen scaffolds. Applied in dental prosthetics and management of bones. Supports reconstructions of tooth trauma.	[22]
β-Tricalcium Phosphate (β-TCP)	Natural sources like bone or synthetic (Sol–gel method).	Porous, biocompatible, resorbable.	Calcium phosphate (Ca_3_(PO_4_)_2_).	Bone regeneration and repair. Enhances scaffold strength and degradation control. Used in periodontal tissue engineering and alveolar bone repair.	[23,24]
Poly(lactic-co-glycolic acid) (PLGA)	Synthetic polymer (lactic and glycolic acid).	Biodegradable, flexible, tunable mechanical properties, low toxicity, form porous structure.	Amphiphilic nature, biodegradable, biocompatible.	Composite collagen scaffolds. Controlled drug and growth factor release. Soft tissue repair.	[25]
Polycaprolactone (PCL)	Synthetic polymer (petroleum-based).	Biodegradable, flexible, high mechanical strength, slow degradation.	Aliphatic polyester, biodegradable, non-toxic.	Used for long-term scaffolding applications. Supports bone and periodontal ligament regeneration. Commonly blended with collagen.	[26]
Polyethylene Glycol (PEG)	Synthetic polymer.	Hydrophilic, forms hydrogels, can be crosslinked, enhances mechanical properties.	Ethylene glycol monomer, non-immunogenic, used for modifying the surface of scaffolds.	Used to improve scaffold mechanical properties. Can form hydrogels to support cell delivery and growth factors. Tissue engineering applications.	[27,28]
Genipin	Derived from gardenia fruits.	Crosslinking agent, biocompatible, enhances mechanical stability.	Crosslinker for proteins, aldehyde-containing molecule.	Used in collagen scaffolds to improve mechanical properties. Reduces degradation rate of collagen scaffolds.	[29]
Growth Factors (BMP, VEGF, FGF)	Naturally occurring proteins in the body.	Protein molecules, bioactive, water-soluble.	Regulate cell growth, differentiation, and tissue repair.	Delivery in scaffolds to promote cell differentiation and tissue healing. Used in periodontal tissue regeneration and alveolar bone healing.	[30]
Nanocellulose	Derived from plant-based materials.	High tensile strength, biodegradable, hydrophilic, high surface area.	Cellulose fibrils, biocompatible, supports cell adhesion.	Can be used for reinforcing collagen scaffolds. Potential for enhancing mechanical properties of scaffolds while being environmentally friendly.	[31,32]
Bioactive Glass	Naturally occurring minerals, synthetic.	Hard, highly bioactive, osteoconductive, forms a bond with bone tissue.	Silicate-based glass, reacts with body fluids to form hydroxyapatite.	Bone regeneration scaffolds. Enhances mineralization in dental tissue engineering. Used in dental implants and alveolar bone healing.	[33]

## 3. Collagen-Based Biomaterials in Dental Tissue Engineering

Collagen is a significant protein in the extracellular matrix and has long been considered useful for dental tissue engineering because it is biocompatible, biodegradable, and shows bioactivity. Most importantly, cell repair and regeneration are supported by collagen-based biomaterials that help cells infiltrate, divide, and develop in tissues.

### 3.1. Types of Collagen-Based Matrices

Collagen-based scaffolds are flexible and are similar to the original extracellular matrix (ECM), which is why they are so widely used in dental tissue engineering. These scaffolds usually fall into one of three categories—pure collagen, composite collagen, or crosslinked collagen—and each has its own set of chemical and biological traits, as well as practical uses. To enhance dental scaffolds, one must study the unique traits and scientific reasons behind every category.

#### 3.1.1. Pure Collagen Scaffolds

Usually, pure collagen scaffolds are taken from cattle, pig, or human tissues for their biocompatibility and gentle reaction after scrupulous cleansing. Many properties such as hydrogels, sponges, membranes, and disks can be created from collagen using either freeze-drying or gelation, and its original triple-helical and fibrillar structure is always kept intact. Pure collagen scaffolds are useful because they very closely resemble the native dental ECM’s biochemicals and mechanics, creating an excellent environment for cells to adhere, migrate, and differentiate. Such sequences as RGD inside the ECM of periodontal and periosteal tissues aid the connection of cells to integrins and enhance signaling, which are vital for the correct choice of odontogenic, osteogenic, or fibroblastic type cells [34,35]. Using hydrogels, stem cells and agents can be delivered inside the body in a minimally invasive way, while sponges and membranes are used to create structures that guide tissue growth (GTR). Although good, the scaffolds only last a short time and are not strong, so they are not usually suitable in pressure-bearing places like alveolar bone or regions near the periodontal ligament. As there are limits to mechanical properties, additional efforts are required to boost function and slow down wear and tear.

#### 3.1.2. Composite Collagen Scaffolds

Composite matrices that use components such as bioactive ceramics, synthetic polymers, and biofunctional molecules have been developed to deal with the problems of pure collagen scaffolds. The intention is to improve the strength, biological functioning, and durability of the scaffold without losing its good interaction with cells. Adding hydroxyapatite (HA) and β-tricalcium phosphate (β-TCP) gives ceramic fillings the ability to reproduce minerals in bone and dentin, as well as assists in the formation of new bone tissue [36]. The presence of these cells in the collagen matrix significantly enhances bone strength, aids in the attachment and growth of osteoblasts, and helps in the formation of healthy mineralized tissue used for alveolar bone regeneration and supporting the periodontal tissue.

To improve the mechanical properties, how quickly the scaffold breaks down, and the scaffold architecture, blends of PLGA, PCL, and collagen are used. Such polymers strengthen materials, allow equipment to bear loads, and can deliver growth factors or drugs over time when needed. Because of composite design, the scaffold can be adjusted to provide mechanical support similar to the periodontal ligament [27,37]. Furthermore, by combining different scaffolds, it becomes possible to add bioactive molecules or antimicrobials which help the scaffold’s functions and protect against infection while healing takes place. The layered structure of composites helps to imitate nature and offers more chances for dental tissue replacement.

**Figure 2:**
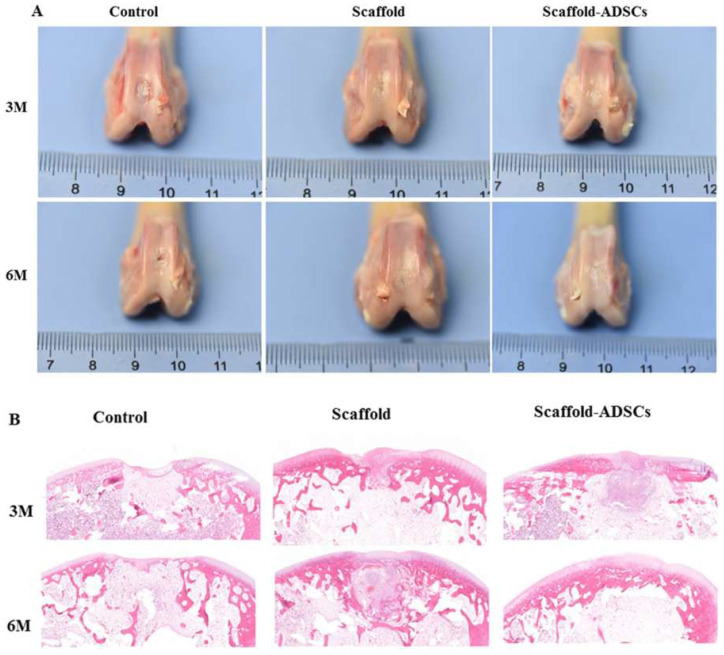
In vivo, Col/SF scaffolds facilitate cartilage repair. (**A**) Macroscopic appearance of specimens obtained at 3 and 6 months after operation. (**B**) At 3 and 6 months, repair cartilage was stained with H&E [38].

In this research, the authors explore the property of ultrasonically treated composite scaffolds (collagen/silk fibroin (Col/SF)) as a cartilage regenerator. Mechanical tests were conducted on the scaffolds which had different Col/SF ratios, i.e., 7:3, 8:2, and 9:1, to test their biosafety and biological activity applied via scanning electron microscopy and Fourier-transform infrared spectroscopy. A 7:3 ratio proved to be effective in maintaining or supporting a process of chondrogenic differentiation of stem cells of adipose tissue in vitro. In vivo studies conducted by the team based on New Zealand rabbits with full- thickness articular cartilage defect revealed that the Col/SF scaffolds were successful in triggering osteochondral regeneration, shown through macroscopic, histological, and immunohistochemical analyses. Figure 2 denotes an idea of how Col/SF scaffolds can be repaired using ultrasound [38].

#### 3.1.3. Crosslinked Collagen Matrices

By using crosslinking, it becomes possible to strengthen the collagen scaffolds and raise their durability against enzymes. By modulating the network of collagen with pooled techniques, it is possible to increase the life of the scaffold and carefully control when the scaffold will break down. Compounds such as EDC, NHS, genipin, and glutaraldehyde can form strong connections among collagen fibrils to make the scaffold stronger and less damaged by proteolytic enzymes [39]. These physical techniques, for example, UV irradiation, dehydrothermal heating, and microwave applications, offer fibrillar stabilization by forming weak links without using dangerous chemicals.

Properly controlling how densely crosslinks are formed ensures infiltration of cells, a good pore structure, and biocompatibility; otherwise, either more or less crosslinking leads to setbacks, so some balance is necessary. Efforts are currently being made to improve collagen matrices with enzymatically sensitive bonds and response to cues from their local surroundings. For cases where the scaffold is needed for a long time and carries a lot of pressure, such as in the periodontal ligament and alveolar bone, crosslinked collagen matrices are especially helpful [40]. With an extended lifespan, ECM keeps supporting cells and facilitates gradual changes to ensure the creation of hard tissues until the animal reaches maturity.

Selecting among pure collagen scaffolds, composite scaffolds, and crosslinked collagen matrices depends on what is needed mechanically, biologically, and clinically for the dental tissue. Unmodified collagen scaffolds give the best compatibility and activity to grow soft tissues, composites provide both mechanical assistance and extra features for hard tissues, and matrices that are crosslinked control their breakdown and ensure firmness [41]. Improvements in materials science and fabrication technology are improving these collagen-based biomaterials, helping to develop future dental scaffolds that blend structure, biology, and effective tissue regeneration.

**Figure 3:**
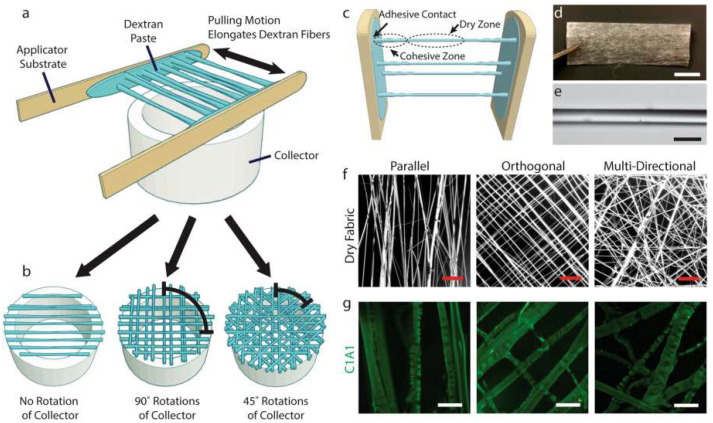
Dextran matrices serve as templates in the formation of ordered collagen fiber networks. (**a**) Templated assembly of the networks of collagen fibers is defined by dextran fabrics. They are created by stretching dextran paste, in a viscous state, between the substrates. (**b**) Fiber orientation is manipulated with the rotation of hollow collector. (**c**) Fibers extend with the balancing of adhesive and coherent forces when they are drying. (**d**) The dried fabrics are durable, workable, and can be cut. (**e**) Individual fibers are uniform, and they are simple to gather together. (**f**) Fabrics may display a number of fiber configurations. (**g**) When dextran is rehydrated, a collagen network is left that assembles together without any intervention, as demonstrated by immunofluorescence [42].

Figure 3 shows that novel existing techniques of preparing collagen-based biomaterials have difficulty in easily aligning microstructural-level collagen fibers, which is fundamental in cell recognition and mechanical performance. Even though fiber-based collagen biomaterials have received some popularity, the existing methods of production could not be applicable in large-scale manufacturing. The new method of fabrication is presented as a template of dextran that allows the effective production of the desired product collagen fiber size. The technique allows myoblasts to adhere and orientate within a 2D collagen fiber network and allows them to differentiate when entrapped in non-cell-attached hydrogel [42]. Such simplicity of production and versatility of this technique allows opportunities to be found in more advanced in vitro tissue models and regenerative medicine applications.

### 3.2. Fabrication Techniques

Making collagen-based scaffolds for dental tissue engineering is key to shaping their properties, their biological reactions, and their overall clinical benefit in various dental procedures. New techniques have been introduced to make use of collagen’s natural activities in a way that improves its strength and durability and helps create scaffolds that resemble the actual extracellular matrix needed for dental regeneration [43].

#### 3.2.1. Freeze-Drying (Lyophilization)

Freeze-drying prepares collagen sponges that have many tiny spaces for cells to reach, nutrients to diffuse, and blood vessels to grow through. A water collagen solution is first frozen and then placed under vacuum, so the ice “sublimates”, leaving a three-dimensional network. Manipulating the rate at which samples are frozen and changing the collagen level help to determine the size and connections of the pores. The scaffolds formed are hydrophilic and swell easily, both of which help to keep a hydrated microenvironment. Freeze-dried collagen sponges are not very strong, which means that after making them, they usually have to be crosslinked or reinforced with different materials for dental repair purposes [44,45].

**Figure 4:**
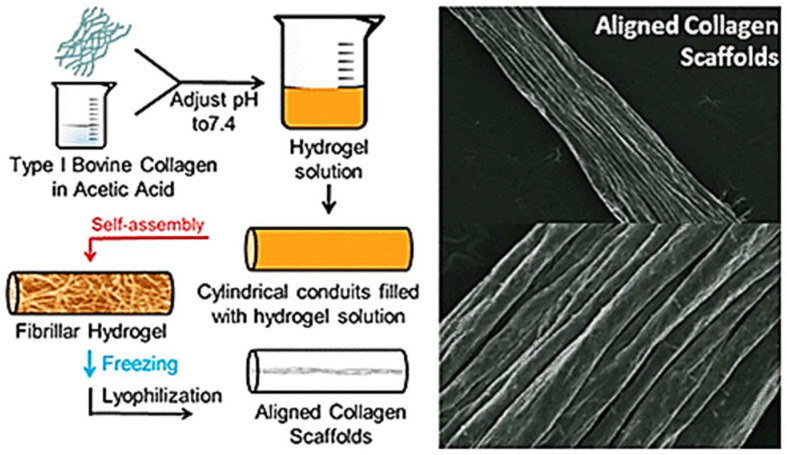
Analysis of morphology of collagen scaffolds. Optical images of scaffolds having different diameters (6350 Âµm, 4762 Âµm, and 3175 Âµm). SEM with straight topography of the surface, consistent straightness in all the length of the scaffold, and cross-sectional appearances with porous insides. In unassembled collagen scaffolds, in the fibrillar collagen in low-aspect-ratio vessels, there was no preferential alignment. All of the samples were synthesized at 2.0 mg/mL concentration of collagen, frozen at −80 °C, and visualized in the dry form [46].

Mimicking of native tissue architecture, i.e., muscle, nerve, and ligaments, where matrix and cellular orientation is fundamental, requires aligned collagen scaffolds. Those conventional techniques to produce aligned collagen scaffold can be time consuming or necessitate special equipment. However, in comparison, an easy, high-speed method with minimal steps was created whereby the collagen becomes a fibrillar hydrogel inside of the cylinder tube that is then frozen and lyophilized to create highly aligned scaffolds. It is also dependent on the starting fibrillar network and on the aspect ratio of the vessel employed [46]. Diameter of scaffolds depends on the concentration of collagen and diameter of conduits, and the dimensions of aligned features depend on conduit diameter and liquid nitrogen temperature of the freezing process. Both the rat dermal fibroblasts and axons of chick dorsal root ganglia have aligned morphology after being seeded on these scaffolds and can, therefore, be used in tissue engineering, where directed cellular alignment is essential (Figure 4).

#### 3.2.2. Electrospinning

Collagen scaffolds made using electrospinning copy the fine structure with fibrillar alignment, which is needed to assist cells in the periodontal ligament and dental tissues with their adhesion, movement, and differentiation. A high-voltage electrical field is used to make a solution of collagen squirt out finer-than-nanometer sized fibers that lie on the collector surface and form the scaffolds. Because electrospun collagen matrices feature a big surface area-to-volume ratio and many pores, there is better support for cell–material interaction and mechanotransduction [47]. In addition, bioactive substances, ceramics, and synthetic polymers can be mixed into composite scaffolds made by electrospinning, giving them better strength and a specific timeframe for degradation.

**Figure 5:**
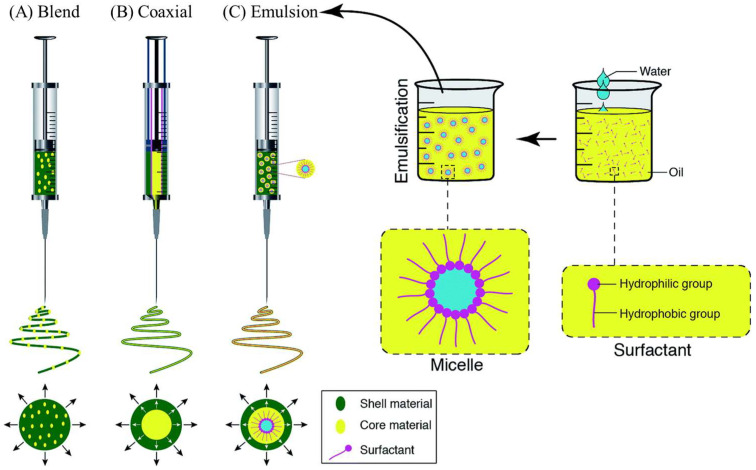
Schematic representations of the loaded spinneret with a bioactive agent to represent (**A**) blend, (**B**) coaxial, and (**C**) emulsion electrospinning [48].

Three electrospinning processes, including blend, coaxial, and emulsion, enable the addition of active agents to the inner part or the surface of nanofibers. The blend method results in the even distribution of the active agent within the fibers, and the coaxial and emulsion methods will result in the core/shell type of fibers. Figure 5 depicts the vast morphological differences between the fibers made using these methods [48].

#### 3.2.3. Three-Dimensional (3D) Bioprinting

Three-dimensional bioprinting makes it possible to precisely control the structure, porous design, and placement of cells within the tissue construct, which allows the creation of custom replacement tissues that fit the individual’s dental anatomy. By combining collagen hydrogels, stem cells, and bioactive substances into bioinks, the bioprinting methods (such as extrusion-based, inkjet printing, or laser printing) accurately form structures with tiny details layer-by-layer. By using this system, it becomes possible to control the movement of cells and biomolecules, supporting both cell life and their functions at the site of transplant [49]. Furthermore, using CAD and imaging tools in design improves the ability to customize products and reproduce them. Even if rheology and mechanical integrity are a challenge, further advancements in bioinks are expanding 3D bioprinting in the field of regenerative dentistry. On top of these engineering benefits, there are also biological ones, which collagen itself provides. These have their own RGD (arginine–glycine–aspartic acid) sequences which facilitate cell adhesion, proliferation, and migration, and this is vital in successful tissue regeneration. This kind of low immunogenicity and perfect biocompatibility of collagen allows it to be allogenic and xenogenic. It can be gradually replaced by host tissue due to its natural biodegradability increasing the speed of normal healing without eliciting any adverse effects. Further, the binding and stabilizing of growth factors with collagen enhances their local bioavailability and tissues remodeling and vascularization.

**Figure 6:**
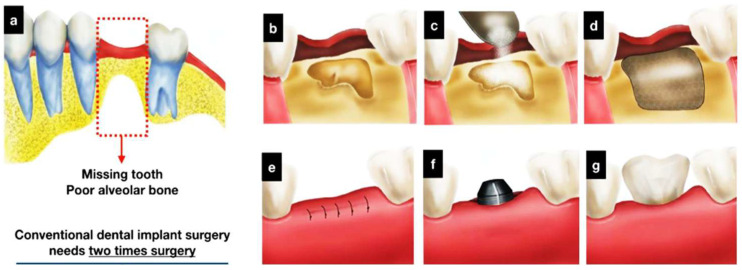
Schematics of a conventional dental implant procedure and a conceptual procedure involving the proposed hybrid scaffold. (**a**) Flow chart diagram of a typical dental implant procedure with the described hybrid scaffold. (**b**–**d**) The process of alveolar bone regeneration (requires first surgery). (**e**–**g**) Insertion of a metal fixture and artificial tooth (requires second surgery) [50].

A new example of hybrid dental combination material in dental implant applications includes a three-dimensional (3D) frame of polycaprolactone (PCL) photopolymerized by a 3D printer with osteoconductive ceramics such as hydroxyapatite (HA) and beta-tricalcium phosphate (beta-TCP) to improve alveolar bone regeneration. The scaffold minimizes numerous surgical treatments, as bone grafts and implant maker insertions could be performed at the same time. Investigation and in vitro studies validated the high porosity, microstructural interconnectivity, and osteoconductivity of the manufactured scaffold. Figure 6 shows that Saos-2 cell experiments indicated better cell proliferation and more alkaline phosphatase activity than the scaffolds used in control [50]. It implies a possible benefit to decrease the cost of treatment and decrease the time period of dental implant operations.

#### 3.2.4. Self-Assembly and Gelation

Self-assembly and the gelation process help generate injectable collagen hydrogels that can store DPSCs, growth factors, and various bioactive agents for easy and safe placement within the patient’s mouth. By using changes in pH or temperature, the collagen in these hydrogels is stimulated to create fibrils, so the formed materials are safe for use in the body and provide a similar environment to ECM. The rate and type of gelation, as well as the mechanical characteristics, can be modified by varying the collagen amount, salt concentration, and which crosslinker is used. Hydrogels made from injectable collagen help cells spread evenly, interact better with the matrix, and slowly release factors that support healing of pulp-like tissue and periodontal tissues [51,52]. But, due to limited strength, these materials must be strengthened by relying on structures that add more support or include special composite materials in the construction.

The microstructure, strength, and biological function of the scaffold are adjusted for every dental application using different fabrication techniques. The use of freeze-drying and electrospinning along with 3D bioprinting and self-assembling hydrogels in scaffolds gives them features similar to real living tissue [53]. The new approaches in fabrication address collagen’s known issues, increase communication between the material and cells, and contribute to the success of collagen in dental tissue engineering trials.

**Figure 7:**
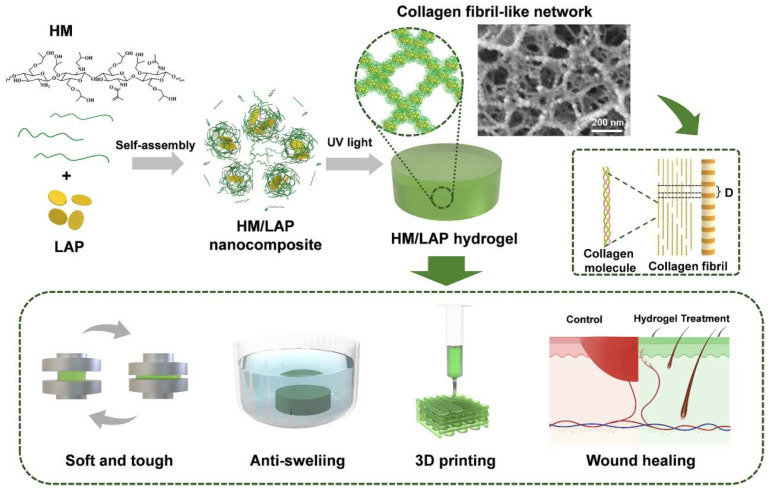
Schematic illustration of the preparation and properties of collagen fibril-like HML nanocomposite hydrogels in tissue engineering [54].

Figure 7 shows how self-assembled nanoparticles have been used to come up with injectable hydrogels that resemble collagen fibrils, thereby increasing the rate at which the wound-healing processes take place. To achieve this, self-assembly-based crosslinked hydrogel with nanoparticles was prepared by the combination of methylacrylyl hydroxypropyl chitosan (HM) and laponite (LAP) followed by crosslinking (via photo-crosslinking). The structure has low rigidity and a high compressive strength combined with antiswelling capabilities with a swelling ratio of 1.07 in PBS. The hydrogels are printable into intricate shapes as a 3D print and possess outstanding biocompatibility and biodegradability. In vitro testing indicated an effective movement of fibroblasts and faster development of blood vessels, representing a perfect manifestation of regenerative medicine [54,55,56]. The properties and biodegradability of the hydrogel mean that it is a good candidate to use in tissue engineering materials such as wound dressings.

### 3.3. Physical and Mechanical Characteristics of Collagen-Based Scaffolds in Dental Tissue Engineering

The success of collagen scaffolds in GTR within the oral cavity is largely affected by their physical and mechanical features. Because native collagen fibrils are organized in a well-planned manner on the nanoscale, micron scale and millimeter scale, they ensure that tissue integrity is maintained regardless of the active forces placed on them. Collagen can assemble at the nanoscale into fibrils, held by hydrogen bonds and with characteristic repeating Gly-X-Y amino sequences, and it helps form the major framework that allows bones to stay strong [43]. Fibrils, in turn, aggregate into fibers and structures, creating the ECM important for tissue structure, biomechanics, and repair. While lysyl oxidase helps produce enzymatic crosslinks, they easily form in the collagen matrix of natural teeth.

Even with their benefits, pure collagen scaffolds are mechanically less strong than dentin, cementum, and alveolar bone. Unmodified collagen hydrogels or sponges have very low elastic modulus and ultimate tensile strength, which means that they cannot handle the chewing and pressure forces in the mouth. Its good ability to hold water and its porous structure, which are valuable for nutrient and cell movement, lead to collagen having a low resistance to compression and a tendency to change shape when compressed [57]. Many studies have been conducted to improve the mechanical strength of collagen-based biomaterials through several methods:

Composite formation: The use of hydroxyapatite (HA) and β-tricalcium phosphate (β-TCP) in composite materials makes the structure similar to the native bone and increases its strength and ability to carry loads without losing bioactivity. They resemble the link between bone and collagen, which supports bone growth and mineralization.

Chemical and physical crosslinking: Using EDC/NHS, genipin, UV, and dehydrothermal processes helps stabilize collagen fibrils by creating covalent bonds which reduces their breakdown by enzymes and strengthens them. Still, it is necessary to control the crosslinking density, as too much crosslinking interferes with cell infiltration and their health.

Synthetic polymer blends: Collagen blended with biocompatible synthetic polymers such as PLGA, PCL, and PEG can produce hybrid scaffolds that can be adjusted to support dental loads, degrade at the proper time, and remain strong.

Advanced fabrication techniques: The use of electrospinning allows the alignment in collagen nanofibers that looks like the PDL’s unique arrangement and supports the right mechanical behaviors and cell function. Because of freeze-drying, scaffolds have porous structures that evenly combine strength with space for blood vessels to grow and connect [58].

The physical features of collagen scaffolds are characterized by measuring tensile strength, Young’s modulus, compressive modulus, elongation at break, porosity, and how quickly they swell, and all these play a role in how well the scaffold will perform in the body. The mechanical features of the materials need to serve the right tissue; for example, alveolar bone scaffolds require extra stiffness, not flexibility like the PDL types. Besides this, the mechanical features of collagen scaffolds alter as the scaffolds break down and the tissue remodels, so the scaffold architecture and chemical elements must be closely monitored to maintain their sturdy structure all through the regeneration process. Basically, because native collagen itself has a strong structure that supports the tissue’s function, when collagen is used as a scaffold it requires being supported further through crosslinking and new fabrication innovations. Because of these improvements, collagen-based biomaterials resemble the mechanical features of minerals more closely in dental tissues, which greatly lowers the risk of failure during their use in dental regeneration.

## 4. Applications in Dental Tissue Regeneration

### 4.1. Pulp Regeneration

Restorative and regenerative endodontics consider pulp regeneration a crucial field because it aims to renew the vitality, use, and structure of the tooth’s nerve chamber when injured or sick. The main part of this method is collagen-based scaffolds that are similar to natural tissue and help cells grow, multiply, and develop into healthy tissue. Most collagen scaffolds, containing mainly type I collagen, have the same structure as the organic matrix in pulp tissue and so are biocompatible, water-friendly, and bioactive. They enable the transport and delivery of DPSCs which can alter into odontoblasts involved in dentin formation [59]. Collagen combined with certain important growth factors known as BMPs and VEGF is found to support the growth of new blood vessels and improve nutrition in the restorative process of the pulp.

Experiments in the lab and in animals have consistently suggested that scaffolds made of collagen and DPSCs, plus osteo/angiogenic factors, help odontoblasts shift to dentin formation, as shown by increased expression of both dentin sialophosphoprotein (DSPP) and dentin matrix protein 1 (DMP-1). By making the scaffold porous, using approaches like freeze-drying and electrospinning, more cells and new blood vessels can enter in, so it can form a functioning, vascularized tissue like normal pulp. In addition, injectable collagen gels provide a simple delivery route that allows the gel to fill any root canal shape. The gel hardens where needed, properly spreads substances, maintains the health of living cells, and allows the site to progress naturally. As the scaffold made by native collagen is weak and can be digested quickly by enzymes, using crosslinking or added composite substances is required to keep it stable during important stages of pulp restoration [60].

New trends in pulp tissue engineering involve combining collagen with nanomaterials, developing sustained ways to provide growth factors and adding gene therapy to help improve the body’s natural healing process. They solve the prior issues of fast breakdown of scaffolds and low rates of proper blood supply which stopped these innovations from being clinically useful. All in all, collagen-based scaffolds are important in regenerating dental pulp as they give stem cells and growth factors a biologically active base that helps form useful tissue with repairing capabilities in the dentin layer. With advances in material science and biofabrication, their improvement is predicted to solve existing problems and make regenerative endodontics more predictable and reliable.

### 4.2. Periodontal Tissue Engineering

Periodontal tissue engineering is now an important focus of regenerative dentistry that aims to recover the supporting system for teeth by building back the periodontal ligament (PDL), cementum, and alveolar bone. Damages to the tissues, usually from periodontitis, physical injuries, or surgical operations, cause teeth to become less steady, reduce chewing ability, and may lead to tooth loss. While traditional periodontal approaches stop bacterial growth and reduce symptoms, they rarely help the damaged tissue in the mouth regenerate. Therefore, advanced materials aim to encourage new tissue formation and its function [61]. Due to their ability to be used in the periodontium without harm, to trigger various biological responses, and to imitate natural ECM structure, collagen-based biomaterials now play an important role in GTR. Frequently, GTR uses collagen membranes to cover a surgery site which prevents gingival and epithelial cells from going there while allowing periodontal ligament cells and osteoprogenitor cells enough time to move in. For the periodontal attachment apparatus to be restored, it is important that the PDL fibers, cementogenesis, and alveolar bone are correctly reformed when cells repopulate.

Cell adhesion and multiplication and differentiation of periodontal ligament fibroblasts, cementoblasts, and osteoblasts are promoted in collagen scaffolds by the ideal configuration of collagen and its network structures. Because collagen is hydrophilic, it allows nutrients and oxygen to move easily which helps keep cells alive and active in the regenerative niche. Still, membranes made of pure collagen are very compatible with the body, yet they are not very robust and break down quickly with enzymes, so they may not stand up well to larger or higher-loaded areas inside the mouth [62]. Composite collagen–ceramic scaffolds are used to improve these limitations by mixing hydroxyapatite (HA) and β-tricalcium phosphate (β-TCP) into the structure. Such composites are like the mineral part of alveolar bone and cementum found in the mouth, raising the scaffold’s stiffness and its ability to promote new bone growth. The presence of minerals in the scaffold gives support to structures and also aids the attachment and multiplication of osteoblasts that are important for alveolar bone and cementum growth.

The use of new techniques like electrospinning and 3D bioprinting gives scientists the ability to control scaffold microstructures so they can recreate the specialized alignment of PDL collagen fibers. This detail in architecture makes sure that cells line up properly and support new extracellular matrix which helps restore effective PDL with good strength [63]. In addition, using certain growth factors such as BMPs, VEGF, and PDGF during the manufacturing of collagen-based scaffolds potentiates angiogenesis, osteogenesis, and cementogenesis, helping the tissues recover quickly. Injectable hydrogel containing both PDLSCs and special molecules offers a simple and safe treatment that provides support for cells, their growth, and tissue repair in different parts of irregular periodontal defects. These hydrogels help the formation of blood vessels and regulate inflammation which are important for the health of the periodontium.

In periodontal surgery, collagen membranes have proven to be highly effective, resulting in better attachment around the tooth, shallower pockets, and an increase in bone growth. Composite collagen–ceramic scaffolds are being studied more frequently for use in preserving and regenerating alveolar ridge before dental implant placement, proving their relevance in biological science. Regardless of what has been achieved, problems such as variability between different batches, issues related to immune responses, and damage to scaffolds caused by sterilization are still obstacles to broad use in medicine [64]. Improvements in the future may see collagen be used that can adjust its degradation and let out bioactive molecules in reaction to the situation in the gingival tissue. Adding bioactive glass or silver nanoparticles together with nanotechnology might make dental implants stop bacterial growth and encourage new bone formation in the mouth. Overall, various collagen-based materials, including simple membranes and more complex collagen–ceramic constructs, play a key part in regenerating periodontal tissues. Being able to copy the chemicals and shape of the native environment, as well as having increased strength and properties, these scaffolds help treat the PDL, cementum, and alveolar bone through real tissue regeneration.

### 4.3. Alveolar Bone Regeneration

Dental tissue engineering depends on alveolar bone regeneration to rebuild the strength and purpose of teeth damaged by periodontal disease, injury, or taken out by extraction. As they are similar to natural tissues and active in the body, collagen-based biomaterials are widely used as scaffolds to help alveolar bone recovery. Nonetheless, as native collagen does not have enough strength and can be rapidly broken by digestive enzymes, it must be optimized for the various needs and challenges of repairing alveolar bone [65].

Hence, using hydroxyapatite (HA) and β-tricalcium phosphate (β-TCP) ceramics as fillers within collagen matrices is known to be highly effective. Their bioactivity makes ceramics like bone, thus providing better mechanical strength and allowing minerals within the scaffold to form faster. As the collagen–ceramic link is very similar to the natural bone matrix, it encourages osteoblasts to attach, multiply, and specialize [66]. Because of this, the extracellular matrix solidifies and mineralizes, allowing for strong bone and blood vessel growth needed for successful bone remodeling in the alveoli.

Hydroxyapatite lets bone cells integrate by guiding the development of calcium phosphate crystals and a continued supply of calcium and phosphate ions that are needed for bone mineralization and changes. If we change the ratio and particle sizes of these ceramics in the collagen composites, can match scaffold stiffness, pores, and the rate of degradation to suit different bone defect locations and healing schedules. Besides giving shape, the collagen content in the ECM protects important bioactive sequences like RGD that enable cells to adhere to the matrix and start the necessary pathways for osteoblast development and ECM formation [67,68,69]. Furthermore, as collagen is hydrophilic, it provides hydration and the necessary nutrients for healthy cells as well as encourages the growth of blood vessels for providing growth factors to the new bone.

When freeze-drying and electrospinning are used, collagen–ceramic scaffolds become highly porous, allowing for better cell penetration, blood vessel formation, and nutrient exchange. The implementation of crosslinking approaches helps enrich a scaffold’s stability, moderate the rate of degradation, and protect the mechanical function throughout the early stages of bone repair. Composite collagen–HA/β-TCP scaffolds have shown hopeful results in both ridge reconstruction and bone defect repair in the periodontium. They serve as a friendly matrix that permits the settlement of osteogenic cells and, at the same time, restricts the growth of soft tissue into such units [70]. Several studies have looked at using scaffolds to transport osteoinductive growth factors like BMP-2 and also stem cells to make regeneration even more effective.

Although many progresses have been achieved, it is still challenging to ensure that scaffolds survive chewing, to control their breakdown over time, and to guarantee that natural collagen is consistent in every batch. Further progress may come with combining new nanoparticles for their antimicrobial and improved osteoinductive abilities, improving the design of scaffolds for individuals using 3D bioprinting, and making collagen composites that can change their work and breakdown based on the local environment. To sum up, the addition of hydroxyapatite or β-tricalcium phosphate to collagen makes the material similar to natural bone and promotes the development, growth, and maturation of new bone.

### 4.4. Enamel Repair (Emerging)

As dental enamel has no living cells, it is not able to fix itself when suffering from caries, erosion, or any sort of trauma. Esthetic dentistry often uses artificial materials that look good but do not possess the same structure, strength, and functional role as the original enamel. As a result, these dentistry solutions do not last or work as well as healthy teeth and can cause new oral issues. Modern advances in dental tissue engineering have proposed collagen, mainly type I collagen, as a promising material to direct the repair of enamel through biomimetic mineralization [71]. Even though there is no real collagen in enamel, the presence of an extracellular-matrix-like collagenous template gives the structure bioactivity and directs the growth and placement of hydroxyapatite crystals just like real enamel grows.

Because collagen has a triple helix, a fibrous structure, and a water-loving nature, it can easily hold calcium and phosphate ions and deliver binding sites for integrins which might moderate the actions of ameloblast or enamel cells included in regenerative treatments. Using collagen-based scaffolds, scientists have made materials containing enamel matrix proteins, peptides, or enamel analogs to support directed mineral growth and form apatite crystals like those in real enamel which have a similar microstructure to native enamel rods. With the use of electrospinning, 3D bioprinting, and self-assembling hydrogels, scientists are now able to produce collagen scaffolds that are shaped to promote growth of enamel tissue [72]. On top of that, collagen scaffolds that are coated with bioactive nanoparticles, fluoride, or peptides like amelogenin have been shown to result in better and quicker mineralization as well as better structure and strength.

Even so, it is challenging to match the structure of enamel and the way it connects to dentin which is needed for proper dental function. Because pure collagen decays fast, making the scaffold composites and carefully crosslinking them is needed to extend its stability during slow mineralization. Moreover, it is necessary to make enamel biomimetic models standard and test them in real clinical environments for these new approaches to work in therapy. To sum up, biomaterials that contain collagen play a key role in building substitute enamel tissue by helping direct the setup of similar mineral structures. Because they can connect the features of enamel’s structure with the cell-assisted repair of bones, they open up new possibilities for gentle and biologically based dental repair. Future advances in enamel regeneration therapy will require conducting studies that consciously integrate materials science, molecular biology, and clinical dentistry.

## 5. Biological Performance and In Vitro Studies

Collagen-based biomaterials depend on their biological properties to be successful in guided tissue regeneration for dental care. By nature, collagen matrices support vital cell processes such as adhesion, multiplication, and changes in dental cell groups like DPSCs, PDL fibroblasts, and osteoblasts. The recognition of collagen by receptors depends on the RGD sequences in its structure which cause the activation of important cellular pathways involved in cell lineage functions. Considerable in vitro studies show that adding bone morphogenetic protein-2 (BMP-2) and fibroblast growth factor-2 (FGF-2) to collagen scaffolds helps cells differentiate faster, speeds up new matrix formation, and supports the growth of blood vessels in the regenerating tissue [40,43]. Furthermore, bioactive modifications are similar to the native signals which team up to encourage the growth of cells linked to odontogenic, osteogenic, and fibroblastic processes.

So that the possibility of infection can be controlled during tissue healing, collagen matrices have been designed to contain both antibacterial peptides and medicinal agents. Using this bacteria-fighting coating helps avoid microbial growth and the formation of film and it allows cells to remain active and deposit extra material for continued healing, without causing harm. The use of antimicrobial molecules becomes important because the oral cavity is home to microorganisms and surgery sites are easily exposed to infection [73]. Strengthening crosslinks is necessary to boost scaffold durability and reinforce the structure, which is important when tissue is being reconstructed and rebuilt.

Using chemical mixtures and ultraviolet rays on collagen crosslinks improves its stability, ensures the scaffold is used longer, and boosts the strength of the collagen. Nevertheless, excessive or poorly optimized crosslinking, as evaluated in the lab, can be harmful to cells, making it harder for them to migrate, grow, and obtain essential nutrients. Therefore, proper adjustment of crosslinking and techniques helps create a structure that offers strength and supports the healthy activity of cells. Together, these studies indicate that collagen-based scaffolds are functional by directing cell functions and providing structural framework which supports the growth of dental tissues [74]. Therefore, experts should continue to work on making collagen scaffolds more effective and less likely to harm the body. With these new advancements, it will be possible to create advanced collagen matrices that supply specific signals, fight bacteria, and offer required strength, which will drive the advances in clinical dental tissue engineering.

## 6. Clinical Applications and Trials

Clinical regenerative dentistry greatly relies on collagen-based biomaterials for their compatibility, useful qualities, and close resemblance to the natural extracellular matrix materials found in oral tissues. Dental tissue engineering covers the use of collagen in various domains like periodontal care, pulp regeneration, and augmentation of bone in the alveolar ridge, with each application relying on a tailored collagen structure [75].

### 6.1. Periodontal Surgery and Guided Tissue Regeneration (GTR)

Collagen membranes have been widely and successfully employed during periodontal surgery to help with guided tissue regeneration. They mainly restrict the growth of epithelial and gingival fibroblast cells into the empty periodontal pockets, which in turn allows periodontal ligament cells, cementum cells, and bone cells to grow and fill the voids. Studies conducted over a long period and clinical trials found lower probing depths, better clinical attachment levels, and more bone under the gingival tissue [76]. Because collagen membranes dissolve on their own, it is not necessary to remove them surgically, which improves patient cooperation and limits possible side effects after the procedure. Yet, as native collagen membranes are easily torn and have variable breakdown rates, it is logical to improve the issue by creating new composite membranes with bioactive ceramics or synthetic polymers, so they can better protect and maintain strength over time in more difficult or stressed repairs.

**Figure 8:**
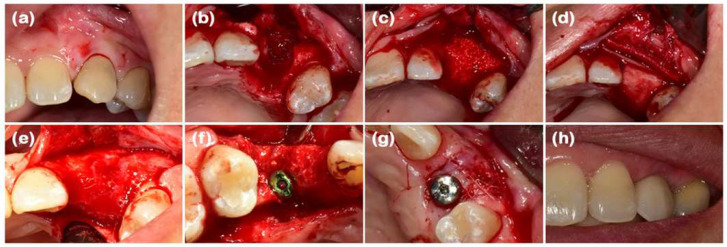
Case 1: MD; (**a**) The pre-extraction maxillary left canine. (**b**) There is buccal bone defect after elevation flap. (**c**) By using DBBM graft, the defect region improves. (**d**) The defect area is clogged with the use of soft/hard collagen membrane and the outer membrane of collagen. (**e**) Entire wound becomes healed and now the bone becomes sufficient. (**f**) To place an implant. (**g**) There is secondary surgery. (**h**) Final prothesis [77].

Collagen membranes have reached a point where clinical evidence shows that these membranes are able to selectively hinder epithelial downgrowth whilst providing the ability to re-populate periodontal ligament cells, cementoblasts, and osteoblasts (Figure 8). The longitudinal studies have proved their therapeutic worth constantly, with probing depth shrinkage of 2–3 mm, clinical attachment level increases of 1.5–2 mm and substantial radiographic bone fill in intrabony defects. Nevertheless, the resorption profile (approximately 4–8 weeks) and limited tensile strength (0.5–3 MPa) of the native collagen membranes limit their clinical capability regardless of the successful union of bony defects. The resultant limitations have necessitated the advancement of next-generation composite membranes with the addition of β-tricalcium phosphate (β-TCP) or polycaprolactone (PCL) reinforcement, displaying even longer rates of degradation (12–26 weeks) and 3–5-fold enhancements in mechanical stability without the loss of biocompatibility.

The development of new methods of preserving the ridges has reached an extreme level in the double-layer alveolar ridge preservation (ARP) technique, whereby a cover layer of collagen matrix works in synergy with underlying barrier membranes to restore both substance and soft tissue loss. The bilayered structure accomplishes this twofold mission: the inner membrane retains graft integrity (with 1.2–2.3 mm narrower horizontal ridge loss than single-layer, wave forms), whereas the outer collagen layer initiates formation of keratinized mucosa. This technique overcomes the typical 40–60% soft tissue contraction that is experienced with traditional ARP, especially when in periodontally compromised extraction areas. Initial CBCT studies support this in complex defects where the augmented ridge size of 6–8 mm allows standard-diameter implants. Randomized controlled trials should be given priority in future research directions to compare the resorption kinetics of different collagen composites (Type I/III vs. crosslinked) as well as histological evaluation of the maturation pattern of neotissue to optimize the effectiveness of this relatively novel regenerative technique [77].

### 6.2. Injectable Collagen Hydrogels

Recent clinical research explores injectable collagen hydrogels as alternative soft scaffolds to help regenerate the dental pulp non-invasively. The dental pulp stem cells (DPSCs) and various growth factors, like VEGF and BMPs, are placed inside the hydrogels to support the repair and regrowth of dental-like tissue and help odontoblast cells develop. As hydrogels can be injected into root canals, they can fit into the curves and turns of a root while keeping the cells alive and active [78,79]. Clinical trials are still running to ensure that collagen-based hydrogel can help recover pulp health and dentin and to guarantee their long-term safety. Even if early studies show valuable results, it remains a challenge to stop the breakdown of scaffolds by enzymes, ensure the scaffold stays strong enough, and support the growth of tissues during the regeneration process.

### 6.3. Composite Collagen Scaffolds for Alveolar Ridge Augmentation

When it comes to alveolar regeneration in advance of implants, composite collagen scaffolds loaded with osteoconductive ceramics are currently being thoroughly evaluated in clinical trials. As a result, having composite scaffolds increases strength, ensures bone cell integration, supports the growth of more bone cells, and helps bone healing through the formation of mineralized matrix. Studies of patients have shown that these procedures encourage alveolar ridge preservation and can be helpful for growing the bone height which supports better implant connection and longer-term results [80]. Because of its biocompatible design and the presence of minerals, the scaffold well mimics the real bone structure. As a result, it permits the easy growth of blood vessels and development of new bone. By using growth factors and stem cells along with tissues, the regeneration of organs is improved. Still, the translation of orthopedic technology to the clinic is slowed by how fast some scaffolds are removed from the body, consistent load management, and creating reliable composite groups [81].

### 6.4. Limitations and Clinical Challenges

Although collagen-based biomaterials have many prospects in medicine, a range of limitations prevents them from being used across the board. It is important to note that when sources of collagen are different from batch to batch, mainly bovine, porcine, or human, the scaffold’s attributes and cell responses may differ too. Due to this variation, the final viral products might still perform erratically, wear down fast, be able to provoke an immune reaction, and can still be a source of disease, even after being filtered and treated. Additionally, the manner in which collagen scaffolds are broken down by enzymes may not keep up with the active healing in the tissue, which could result in their loss earlier than the tissue needs. Even though sterilization is important for safety, it can damage the molecular structure and use of collagen. Immunogenic reactions, which rarely happen, can still affect clinical results in those who are sensitized.

### 6.5. Ongoing and Future Clinical Directions

Efforts in clinical trials and translational research now place emphasis on facing these issues by combining collagen with modern biomaterials such as nanomaterials, growth factor delivery devices, and sensitive, condition-responsive matrices that respond well and biodegrade inside the unique physiological spaces of patients. New techniques in scaffold making, such as 3D bioprinting and electrospinning, allow more accurate anatomical designs and direct cell growth, which boost both the effects of regeneration and accurate predictions for treatment. In addition, treatments using stem cells and gene therapy in collagen-based implants may greatly boost the success of regenerative dentistry. Clinical trials involving different groups of patients and standardized ways to review the materials will be necessary to certify their proper use, value, effectiveness, and safety in dentistry.

## 7. Challenges and Limitations

Even though collagen-based scaffolds are helpful in guided tissue regeneration (GTR) for dentistry, some obstacles still slow down their clinical application. Such limitations are largely caused by the nature of collagen itself, as well as by challenges in obtaining it and using it clinically. Learning about these barriers is necessary for shaping future investigations and the process of scaffold innovation.

### 7.1. Mechanical Weakness

However, naturally produced collagen is not suitable for tough applications including bone regeneration or ligament support because of its sensitive structure. Collagen has an excellent nanoscale strength when it forms the triple-helical fibrils, but this does not provide it with much tensile or compressive strength in pure scaffolds. Because of this deficiency, there is a higher possibility of deformation, issues with chewing power, and failure to support the growth of tissues during early healing stages. Harsh conditions in the body might end in unmodified collagen structures losing their integrity and function, making recovery less likely. Enhancing the strength is vital in tissue engineering, and strategies include using composite materials (such as hydroxyapatite or β-tricalcium phosphate), making mixtures of synthetic polymers (PLGA and PCL), and applying crosslinking approaches.

### 7.2. Batch-to-Batch Variability Due to Natural Sources

Collagen for biomedical scaffolds is most often collected from animal tissues like those of cows, pigs, or even from humans quite rarely. Because it comes from nature, it results in differences in important features like the spread of molecular weights, the amount of crosslinking, how proteins are organized, and residual immunogenic proteins. How old the animal is, the kind of tissue, and the process used to extract and purify scaffolds can all cause a lot of variation in their consistency. Differences in materials make it hard to create uniform standards for strength, degradation speed, and biological responses. This affects compliance with regulations and reproducible results in medicine. In addition, the variability in batches can influence the scaffold’s ability to attach cells, help them multiply, and take on different roles, causing erratic results. Trying new recombinant collagen techniques, rigorous checking, and using enhanced purifiers helps deal with the issue of differences.

### 7.3. Rapid Enzymatic Degradation and Premature Scaffold Loss

Many proteolytic enzymes in the remodeling microenvironment, such as MMPs and collagenases, can break down collagen very easily. Although controlled breakdown of scaffolds is necessary for them to slowly be replaced by the body, the quick degradation of pure collagen matrices usually leads to a loss of mechanical support and structural scaffold too soon before the wound is healed. As a result of the timing delay, cell movement, blood vessel formation, and support tissue development may be impaired. In addition, accelerated breakdown may reduce the membrane’s ability to prevent the penetration of unwanted soft tissues. Several chemical and physical methods (such as carbodiimide, genipin, and UV irradiation) have been developed to affect how quickly scaffolds degrade; still, too much crosslinking might reduce their open structure and may end up being toxic to cells. It is still challenging to ensure that scaffolds last for the right amount of time without occurring toxicity to the body.

### 7.4. Immunogenicity and Biocompatibility Concerns

Despite its biocompatibility linked to its evolution and similar features to the extracellular matrix, some immunogenic reactions to collagen are possible depending on the source, degree of purity, and methods of processing. In certain patients, soluble proteins, telopeptides, or crosslinking agents may bring about local inflammation, an immune reaction, or a foreign body response. Even though this rarely happens, some immune reactions can stand in the way of scaffold integration, cause tissue to become encapsulated in scar tissue, or result in parts of the scaffold being removed by the body. In addition, although zoonotic disease transmissions are minimized by strict sterilization, they could still happen as a possibility. Using recombinant human collagen and improving purification reduce the risks of an immune response, but doctors should always use good caution.

### 7.5. Sterilization Challenges Affecting Collagen Integrity

It is necessary to sterilize collagen scaffolds for clinical use to avoid any risk of infection. Various conventional sterilization procedures usually damage the molecular and organized structure of collagen. Such processes may lead to denaturation of collagen, breakage of fibrils, reduction in bioactive motifs (such as RGD), and changes in how strong the collagen is. Damages to the scaffold affect how it performs, its strength, and its rate of breakdown, and can lessen its ability to regenerate tissue. Still, scientists have yet to design effective sterilization for microorganisms that protects collagen at the same time. Investigation is being carried out on sterilization using supercritical carbon dioxide, low-temperature plasma, and enzymes to find a balance with good performance of scaffolds.

Even though collagen-based biomaterials are similar to natural tissues and are active, they cannot be widely used in surgeries due to being fragile, having batch differences, easy enzymatic breakdown, possible immune reactions, and problems caused by sterilization methods. Overcoming these challenges calls for combining new materials, advanced ways of manufacturing and bonding, strict quality testing, and different sterilization techniques. The creation of special collagen matrices that match the body’s needs may resolve some challenges and boost the effectiveness of tissue regeneration with scaffolds.

## 8. Advances and Future Directions

### 8.1. Functionalization and Composite Development

With different modifications and the use of composites, collagen-based scaffolds have improved a lot to handle toughness and degradation problems. Adding both bioactive glass and silver nanoparticles to collagen matrices has resulted in the materials having antimicrobial properties, decreasing chances of infection and helping with the growth and development of bones. Nanomaterials used in these scaffolds support and stimulate collagen fibers in the scaffolds, increasing scaffold activity while still being safe for the body. Hybrid scaffolds made from PLGA, PCL, and collagen allow scientists to control both the breakdown and the toughness of the scaffolds. Such composites are similar to bone and periodontal ligament tissue, which is very useful in regenerating load-bearing structures. Also, growth factors and antimicrobial peptides can be bonded or included in the material, helping the control of their delivery and supporting wound healing.

### 8.2. Advanced Fabrication Techniques

The use of advanced tools to shape collagen has greatly increased the precision of its architecture and its ability to work in the body. Three-dimensional (3D) bioprinting provides a new opportunity to precisely arrange the scaffold shape, the placement of cells, and the distribution of biomolecules. Such accuracy allows the production of implants that are made to match a patient’s mouth precisely and they can be used in the mouth without any issues. The use of computer-aided Styrofoam planning and collagen-based bioink with stem cells and growth factors inside is at the heart of novel regenerative medicine designs. Electrospinning creates aligned collagen matrices that are like the natural, anisotropic fiber organization found in the periodontal ligament, which improves the biomimicry of the scaffold. Directing cells, structuring ligaments, and improving strength depend greatly on how collagen fibers are aligned. Such scaffolds feature a large surface area and many pores that allow cells to connect, divide, and grow, and, at the same time, allow the use of materials to make them stronger.

### 8.3. Stem Cell and Growth Factor Delivery

Using stem cell biology along with collagen in scaffolds has become very important in enhancing dental tissue regeneration. Adding controlled release systems to collagen matrices supports the steady and targeted distribution of BMP-2, VEGF, and other essential growth factors so that the injured area receives the needed cells, development, and new blood vessels for tissue recovery. The systems make use of membranes, nano-sized particles, or the affinity between drugs and cells to help targeted delivery of growth factors. Gene therapy along with collagen scaffolds offers a fresh opportunity, because gene-activated matrices make endogenous regenerative proteins, enabling the cells to last a long time. Using stem cells from dental pulp and periodontal ligaments with gene delivery tools in collagen matrices could help promote the growth of tissues and their matrices at the site of application.

### 8.4. Personalized and Smart Biomaterials

Enhancing collagen-based dental biomaterials involves the creation of individualized and environmentally responsive scaffolds that adjust to different physiological and health issues. Merging micro-CT, MRI, and computer-aided design (CAD) can allow the creation of custom-built scaffolds that are a perfect fit for the shape of the bone defect and aid in recovery. New smart collagen matrices react to things like pH changes, enzymes, stress, or temperature within the healing microenvironment by changing their breakdown and release of active molecules. Adaptive behaviors support, at the same time, scaffold removal and new tissue growth, leading to better regeneration. Moreover, including biosensors in collagen scaffolds gives real-time data about what is occurring in the area, making it possible to plan accurate and appropriate treatments for each patient.

## 9. Conclusions

The use of collagen-derived scaffold is a radical measure to be applied in dental tissue engineering through the availability of unprecedented biocompatibility and bioactivity in pulp regeneration, periodontal tissues, and alveolar bone. Nevertheless, additional limitations and barriers to clinical translation are due to natural characteristics, including fast enzyme degradation, mechanical instability, and reproducibility over batches. New directions in composite manufacture (e.g., ceramic reinforcements, synthetic polymer mixes), novel crosslinking technologies, and the precision of technologies such as 3D bioprinting have been an advanced solution to overcoming this barrier, allowing scaffold behavior to be developed to a specific purpose (e.g., allowing customizable degradation times and maximum weight bearing). Current developments like smart biomaterials that react to micro-environmental signals, stem-cells-loaded hydrogel, and gene-reactive matrices will transform personalized regenerating therapy. To overcome the dislocation between lab-based innovation and clinically applicable application, research that can be conducted in the future should focus on, first, the development of standardized production processes; second, long-term evaluation of efficacy in vivo; and, third, the possibility of extending production techniques to large-scale application. With the resolution of such issues, the next-generation collagen biomaterials might be able to realize a level of predictable long-term results, eventually rendering dental regeneration a common clinical practice.

## Data Availability

No new data were created or analyzed in this study. Data sharing is not applicable to this article.

## Abbreviations

PDL—periodontal ligament; ECM—extracellular matrix; GTR—guided tissue regeneration; RGD—arginine–glycine–aspartic acid (motif); HA—hydroxyapatite; β-TCP—beta-tricalcium phosphate; PLGA—poly(lactic-co-glycolic acid); BMP—bone morphogenetic protein; VEGF—vascular endothelial growth factor; DPSCs—dental pulp stem cells; Fgf—fibroblast growth factor; DMP-1—dentin matrix protein-1; DSPP—dentin sialophosphoprotein; PCL—polycaprolactone; PEG—polyethylene glycol; MMPs—matrix metalloproteinases; 3D—three-dimensional; CAD—computer-aided design
